# Phenotypic and evolutionary implications of modulating the ERK-MAPK cascade using the dentition as a model

**DOI:** 10.1038/srep11658

**Published:** 2015-06-30

**Authors:** Pauline Marangoni, Cyril Charles, Paul Tafforeau, Virginie Laugel-Haushalter, Adriane Joo, Agnès Bloch-Zupan, Ophir D. Klein, Laurent Viriot

**Affiliations:** 1 Evo-Devo of Vertebrate Dentition, Institute of Functional Genomics of Lyon, ENS de Lyon, CNRS UMR 5242, Université Lyon 1 – Université de Lyon, 46 allée d’Italie, 69364 Lyon cedex 07, France; 2 European Synchrotron Radiation Facility, 71 avenue des Martyrs, CS-40220 F-38043 Grenoble cedex 09, France; 3 Development and Stem Cells Department, Institute of Genetics and Molecular and Cellular Biology, Inserm U 964, CNRS UMR 7104, Université de Strasbourg, BP 10142, 67404 Illkirch, France; 4 Department of Orofacial Sciences and Program in Craniofacial Biology, University of California, San Francisco, CA 94143-0442, USA; 5 Faculty of Dentistry, University of Strasbourg, 8 rue St Elisabeth, 67000 Strasbourg, France; 6 Reference Centre for Orodental Manifestations of Rare Diseases, Pôle de Médecine et Chirurgie Bucco-Dentaires, Hôpitaux Universitaires de Strasbourg (HUS), 1 place de l’Hôpital, 67000 Strasbourg, France; 7 Department of Pediatrics and Institute for Human Genetics, University of California San Francisco, San Francisco, CA 94143, USA

## Abstract

The question of phenotypic convergence across a signalling pathway has important implications for both developmental and evolutionary biology. The ERK-MAPK cascade is known to play a central role in dental development, but the relative roles of its components remain unknown. Here we investigate the diversity of dental phenotypes in *Spry2*^*−/−*^, *Spry4*^*−/−*^, and *Rsk2*^*−/Y*^ mice, including the incidence of extra teeth, which were lost in the mouse lineage 45 million years ago (Ma). In addition, Sprouty-specific anomalies mimic a phenotype that is absent in extant mice but present in mouse ancestors prior to 9 Ma. Although the mutant lines studied display convergent phenotypes, each gene has a specific role in tooth number determination and crown patterning. The similarities found between teeth in fossils and mutants highlight the pivotal role of the ERK-MAPK cascade during the evolution of the dentition in rodents.

The extracellular signal-regulated kinase/mitogen-activated protein kinase (ERK-MAPK) pathway is a central regulator of tooth development. This cascade is typically initiated by the binding of a growth factor to a receptor tyrosine kinase (RTK), which triggers the phosphorylation of successive kinases and culminates in activation of effector kinases and the transcription of target genes (Fig. 1)^1^. The MAPK signalling pathway has been intensively studied by cancer biologists because of its effects on regulation of cell proliferation and survival^1^,^2^, but this pathway is also important throughout mouse embryogenesis^3^. The pathway has been investigated in numerous embryonic processes, including development of the central nervous system and mesodermal derivatives^4^,^5^, skeletal development^6^, and tooth development^7^,^8^,^9^,^10^,^11^.

Tooth development is a well-documented example of ectodermal organ development. It is a tightly regulated process arising from the crosstalk between dental epithelium and its underlying mesenchyme^12^. The signalling networks responsible for properly building the dentition have been heavily investigated, and numerous members of the ERK-MAPK signalling pathway are known to play a role in tooth development. Early studies examined the fibroblast growth factors (FGFs) that activate their receptors (FGFRs), thus triggering the ERK-MAPK phosphorylation cascade^13^,^14^. Subsequent investigations determined that additional components of the cascade were involved in tooth development^8^,^15^,^16^,^17^. An exciting current challenge is to understand the complexity of feedback regulation in this signalling pathway, which can be stage- and/or tissue-specific. In the present study, we compare the phenotype of molar teeth in mice carrying mutations in Sprouty1, Sprouty2, Sprouty4, and *Rsk2* genes, which are involved at various levels in the MAPK cascade (Fig. 1).

The Sprouty (Spry) family of genes encodes general RTK inhibitors^18^,^19^. After stimulation by growth factors, the Sprouty proteins have been proposed to function by translocating to the plasma membrane, where their phosphorylation prevents the formation of an FGFR adaptor complex^20^; however, the biochemistry of the Sprouty proteins is still the subject of much debate. *Spry1* is expressed in both the epithelium and the mesenchyme, with the exception of a cluster of non-proliferating epithelial cells that serve as a signalling centre called the enamel knot. *Spry2* is expressed only in the epithelium adjacent to the dental mesenchyme, including the enamel knot, and *Spry4* is expressed in the dental mesenchyme^7^,^21^. Whereas the morphogenesis of molar teeth in *Spry1*^*−/−*^ mice has not yet been examined, *Spry2*^*−/−*^ and *Spry4*^*−/−*^ mice are known to have abnormal dentition, which sometimes includes supernumerary teeth (ST) located immediately in front of the first lower molar^7^. These supernumerary teeth, which occur at differing frequencies depending on the genetic background^7^,^22^,^23^, are believed to derive from evolutionary vestigial tooth buds that normally undergo apoptosis in wild-type embryos^24^,^25^,^26^. Lagronova-Churava and colleagues (2013) showed that although all *Spry2*^*−/−*^ and *Spry4*^*−/−*^ embryos present a revitalisation of tooth rudiments at ED13.5, only 2% of *Spry4*^*−/−*^ and 27% of *Spry2*^*−/−*^ specimens had a lower ST. However, the role of *Spry1*, *Spry2* and *Spry4* in the development of upper molars is not known, and the adult molar morphology has not been scrutinised in these mutants.

RSKs (90 kDa ribosomal S6 kinases) are effector kinases belonging to the eponymous family of highly conserved serine/threonine kinases^27^,^28^. Out of the four isoforms found in vertebrates, *Rsk2* has been recently demonstrated to be involved in craniofacial development. Indeed *Rsk2*^*−/Y*^ mice display a deformation of the nasal bone as well as diastemal ST which affect mesial parts of both upper and lower first molars^8^. Mutations in *RSK2* have been associated with Coffin-Lowry syndrome (OMIM #303600), a condition characterized by mental and growth retardation along with craniofacial and other skeletal abnormalities^29^,^30^,^31^.

Phenotypic convergence across the ERK-MAPK signalling pathway remains poorly documented. By studying the dental phenotype resulting from mutations in genes located upstream (Sprouty) and downstream (*Rsk2*) of the ERK-MAPK cascade, we address the question of whether an ERK-MAPK signature phenotype exists. To answer this question, we have characterized the molar phenotype in several mutant populations in order to evaluate the distribution of the various changes affecting both upper and lower molar rows. We have also precisely quantified the occurrence of supernumerary teeth (ST) as well as their impact on the other teeth of the row. Finally, we have addressed the evolutionary role of the ERK-MAPK cascade by comparing specific dental traits of mutants with dental traits of other extant or extinct rodents.

## Results

**Figure 2:**
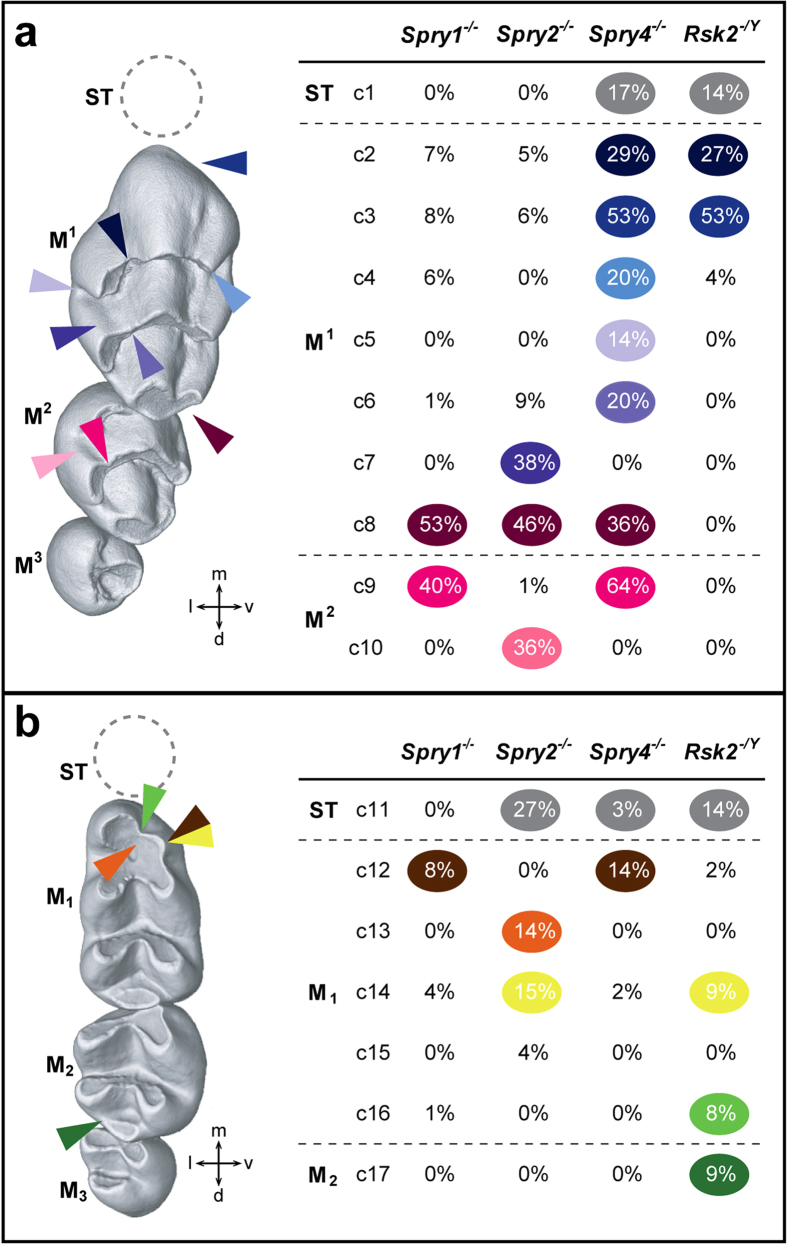
Dental character matrix. (**a**) Upper dental row character matrix (c2–10); (**b**) Lower dental row character matrix (c11–17). On the first column are displayed WT upper (top) and lower (bottom) molar rows, with coloured arrowheads pointing to the localization of the most frequent defects seen in the 4 mutant backgrounds. Each character is listed with the occurrence frequency in all the mutant mice observed. (c1) is the occurrence of a ST; (c2–4) are M^1^ 1^st^ chevron defects, respectively lingual cusp disconnection, straight mesial cusp and absence of the vestibular cusp; (c5) is the presence of an extra lingual cusp between the 1^st^ and 2^nd^ chevrons; (c6) is the disconnection of the lingual cusp of the M^1^ 2^nd^ chevron; (c7) is the occurrence of a lingual crest; (c8) is the occurrence of an extra distal cusp; (c9) is the disconnection of the mesio-lingual cusp of the M2; (c10) is the connection of distal cusps. (c11–17) are defects of the lower mutant postcanine teeth. (c11) is the occurrence of a ST; c(12) is the absence of the mesio-vestibular cusp; (c13) is the display of very symmetric mesial-most cusps; (c14) is the split of the mesio-vestibular cusp; (c15) is the occurrence of an extra mesial cusp; (c16) is the strong disconnection of the mesial cusps of the M_1_; (c17) is the abnormal connection of the 2^nd^ and 3^rd^ lophs of the M_2_.

**Figure 3:**
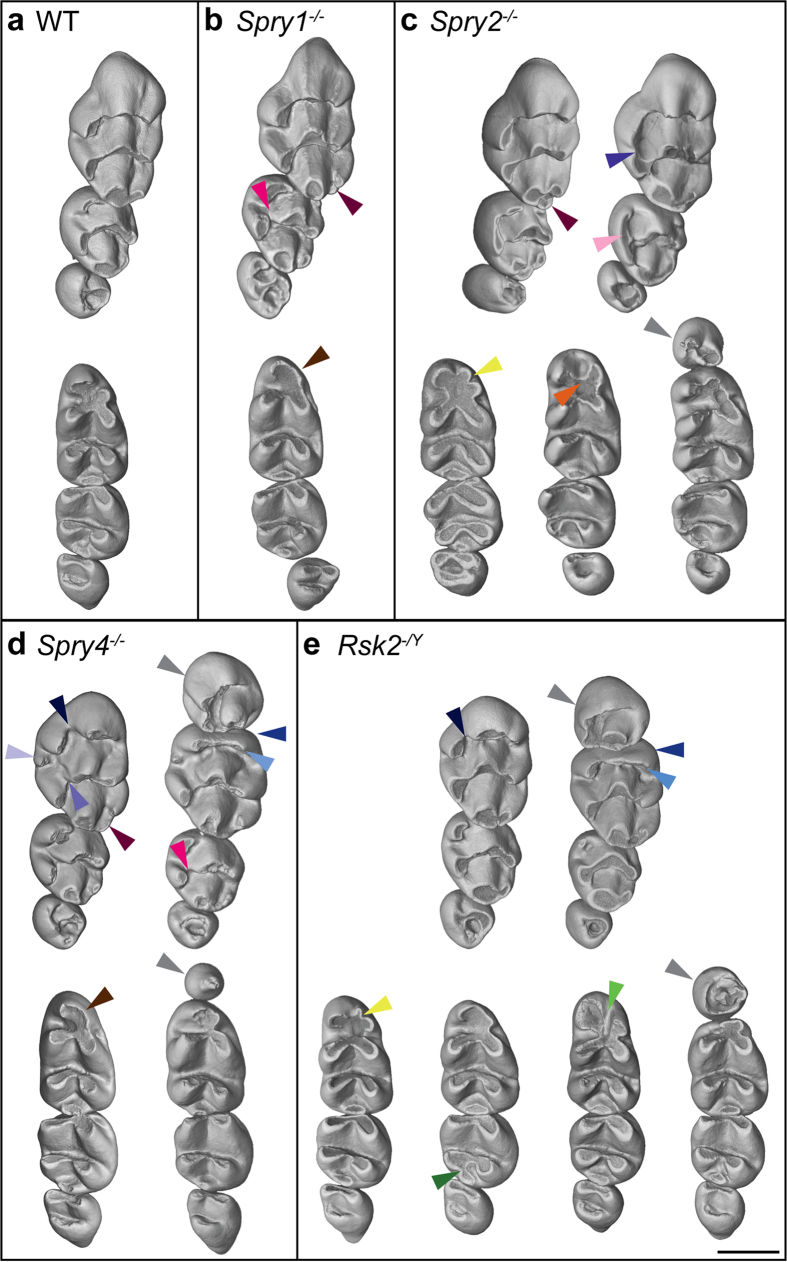
Abnormal phenotypes in the *Spry1*^*−/−*^, *Spry2*^*−/−*^, *Spry4*^*−/−*^ and *Rsk2*^−/Y^ mutant mice. (**a**) WT dental rows; (**b**) *Spry1*^*−/−*^ dental rows; (**c**) *Spry2*^*−/−*^dental rows; (**d**) *Spry4*^*−/−*^ dental rows; (**e**) *Rsk2*^*−/Y*^ dental rows. Coloured arrowheads correspond to the features displayed in the Fig. 2. Note that the dental rows are oriented similarly to what is displayed in Fig. 1. Scale bar: 0.75 mm.

The mouse (*Mus musculus*) is a muroid rodent, and like all the members of this superfamily, mice have a simplified dentition composed only of incisors and molars separated by a long toothless gap called a diastema. Three lower (M_1_ M_2_ M_3_) and three upper (M^1^ M^2^ M^3^) molar teeth are present in each mouth quadrant. The crowns of molar teeth bear a relatively stable number of cusps that are: 8 for M^1^, 6 for M^2^, 4 for M^3^, 7 for M_1_, 5 for M_2_, and 3 for M_3_ (Fig. 2, first column; Fig. 3a). Crowns of upper molars are made of rows of 3 cusps arranged in linguo-vestibular chevrons pointing mesially (except the third one, which is incomplete), whereas crowns of lower molars are made of rows of 2 cusps linked by linguo-vestibular and rather straight crests called lophs (Fig. 2, first column; Fig. 3a). The two mesial lophs of the M_1_ are also linked together by a mesio-distal connection.

### Diversity of the dental phenotypes in Sprouty mutants

**Figure 4:**
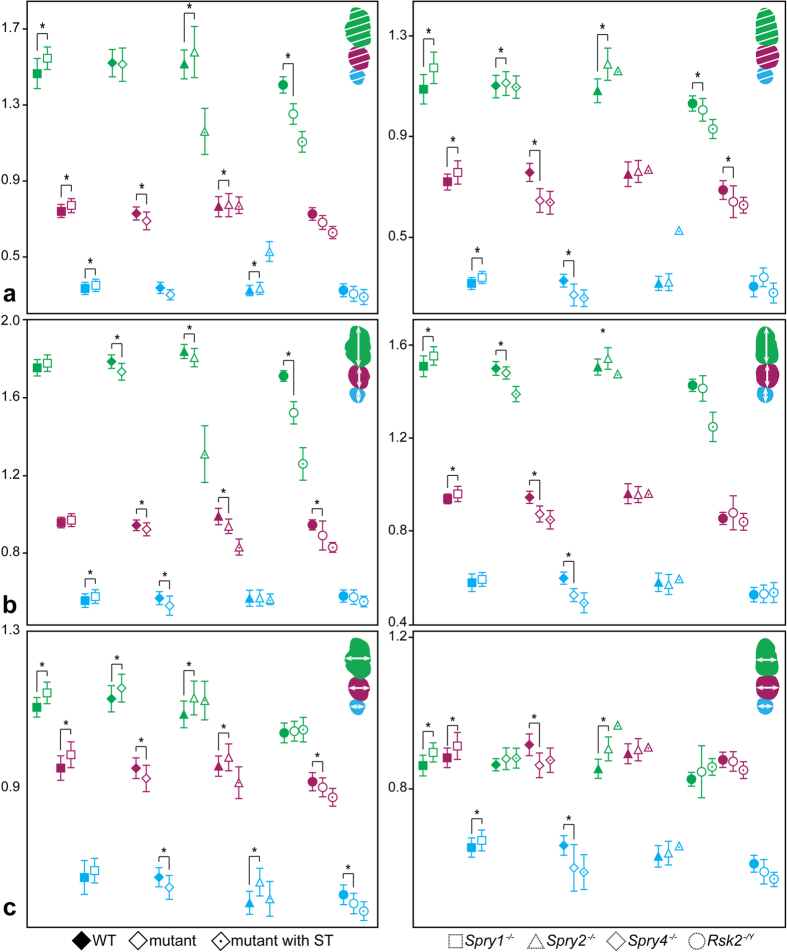
Molar tooth proportions in the *Spry1*^*−/−*^, *Spry2*^*−/−*^, *Spry4*^*−/−*^ and *Rsk2*^*−/Y*^ mutant mice. (**a**) Tooth surface (mm^2^); (**b**) tooth mesio-distal length (mm); (**c**) tooth vestibulo-lingual width (mm). First molars are coloured in green, second molars in pink, and third molars in blue. Filled forms represent the WT for each background, squares represent the *Spry1*^*−/−*^ mutants*, Spry2*^*−/−*^ mutants without (blanked) or with (dot) ST, *Spry4*^*−/−*^ mutants without (blanked) or with (dot) ST and *Rsk2*^*−/Y*^ mutants without (blanked) or with (dot) ST. Significant differences in the tooth proportions are marked with an asterisk (t-test, *p-value* < 0.05).

We examined the arrangement and shape of the postcanine dentition in four populations comprising 25 *Spry1*^−/−^, 50 *Spry2*^−/−^, 50 *Spry4*^−/−^ and 60 WT mice. Area measurements showed that the occlusal surface of molars in *Spry1*^*−/−*^ and *Spry4*^*−/−*^ mice is larger than in the WT mice, whereas the molar occlusal surface in *Spry2*^*−/−*^ mice is smaller than in the WT mice (t test, *p value* < 0.05, Fig. 4a).

The molar teeth of *Spry1*^*−/−*^and WT mice are globally similar in shape, and 51% of the *Spry1*^*−/−*^ dental rows display a WT-like phenotype. The changes are numbered as character# (c#) from the mesial to the distal part of the row, and are mildly to moderately represented in the mutant cohorts. The main defects of the *Spry1*^*−/−*^ postcanine dentition are: (c8) the occurrence of a supplementary distal cusp on the M^1^ (53%); (c9) the disconnection of the mesio-lingual cusp from the first chevron of the M^2^ (40%); and (c12) the absence of the mesio-vestibular cusp of the M_1_ (8%) (Figs 2 and 3b, Suppl. Fig. 1a). The postcanine dentition in *Spry2*^−/−^ mice had stronger differences compared to WT samples, and only 21% of *Spry2*^−/−^ dental rows still display a WT-like phenotype. The main changes of the *Spry2*^*−/−*^ postcanine dentition are: (c7) the connection between the two lingual cusps of the M^1^ (38%); (c8) the occurrence of a supplementary distal cusp on the M^1^ (46%); (c10) the connection between the two lingual cusps of the M^2^ (36%); (c11) the occurrence of a lower ST (27%); and (c13-14) an abnormal shape, number, and/or interconnection of the mesial cusps of the M_1_ (29%) (Figs 2 and 3c). *Spry4*^*−/−*^ molar tooth phenotype is the most variable among the 3 Sprouty mutants. These teeth ranged from a WT-like phenotype (24%) to relatively severe anomalies, especially in the M^1^. The main defects of the *Spry4*^*−/−*^ postcanine dentition are: (c1) the occurrence of an upper ST (17%); (c2–3–4) the presence of lingual cusp disconnection, straight mesial cusp and absence of the vestibular cusp affecting the first chevron of the M^1^ (66% combined); (c5) the occurrence of a supplementary lingual cusp between the first and the second chevron (14%); (c6) the disconnection of the lingual cusp from the second chevron of the M^1^ (20%); (c8) the occurrence of a supplementary distal cusp on the M^1^ (36%); (c9) the disconnection of the mesio-lingual cusp from the first chevron of the M^2^ (64%); (c11) the occurrence of a lower ST (3%); (c12–14) an abnormal number and/or interconnection of the mesial cusps of the M_1_ (16%) (Figs 2 and 3d).

Except for three characters, the dentition of *Spry1*^−/−^ mice thus resembles that of WT mice, whereas *Spry2*^−/−^ and *Spry4*^−/−^ mice show extra cusps and crest disconnections, as well as severe reductions and defects in the mesial parts of the M^1^ (*Spry4*^−/−^ only) and in the M_1_ (rarely in *Spry4*^−/−^, frequently in *Spry2*^−/−^). *Spry4*^−/−^ mice develop ST in both upper and lower jaws, whereas *Spry2*^−/−^ mice only display lower ST. Lower ST have been observed in *Spry2*^−/−^ and *Spry4*^−/−^ molar rows, whereas upper ST only occurred in *Spry4*^−/−^ molar rows. *Spry1*^−/−^ mice thus never develop any ST, and only *Spry4*^−/−^ mutants display ST in both upper and lower tooth rows. These findings suggest a potential relationship between the occurrence of ST and abnormal arrangement of the mesial parts of the first molars.

### Similarities and differences of Rsk2 dental phenotype as compared to Sprouty dentitions

We next compared a cohort of 45 *Rsk2*^*−*/Y^ mice with a cohort of 45 WT littermates. The postcanine occlusal surface area in *Rsk2*^*−/Y*^ mice is smaller than in WT mice (M^1^ and M_1,2_ being significantly smaller, *p-value* *<* *0.05*, Fig. 4a). Although many *Rsk2*^*−*/*Y*^ specimens display relatively severe dental defects, 45% of the examined dental rows display a WT-like phenotype. The main defects of the *Rsk2*^*−*/*Y*^ postcanine dentition are: (c1) the occurrence of a upper ST (14%); (c2–3–4) the presence of many defects on the first chevron of the M^1^ (71%); (c11) the occurrence of a lower ST (14%); (c12 + 14 + 16) an abnormal number and/or interconnection of the mesial cusps of the M_1_ (19%); (c17) abnormal mesio-distal connections between the second and the third lophs of the M_2_ (9%) (Figs 2 and 3e). The frequency of ST occurrence is lower than what has been previously reported^8^, and this may be explained by the examination of a larger sample or by shifts in the genetic background over time.

The comparison of the postcanine dental phenotypes between *Rsk2*^*−*/*Y*^ and the three Sprouty mutant mice shows that *Rsk2*^*−*/*Y*^, *Spry2*^*−/−*^ and *Spry4*^−/−^ mice all develop ST, at varying frequencies. *Spry2*^*−/−*^ mice never have an upper ST, but they frequently have a lower ST (27%). *Spry4*^−/−^ mice frequently have an upper ST (17%), but they rarely have a lower ST (3%). *Rsk2*^*−*/*Y*^ mice develop both upper and lower ST relatively frequently (14%). In addition to developing lower and upper ST, *Rsk2*^*−*/*Y*^ and *Spry4*^−/−^ postcanine dentitions share many similar defects affecting the mesial parts of both the upper and lower first molar. The occurrence of defects on mesial parts of the M^1^ is much more frequent than the occurrence of an upper ST, but the defects in the mesial parts of the M^1^ and M_1_ are also associated with a shortening in length of these teeth (Fig. 4). Finally, *Rsk2*^*−*/*Y*^ mutants do not develop the supplementary distal cusp of the M^1^ common to all Sprouty mutants, nor do they share with *Spry1*^−/−^ and *Spry4*^−/−^ mutants the trend towards having larger teeth than the WT mice.

### The presence of ST impacts both the shape and size of the other teeth in the row

**Figure 5:**
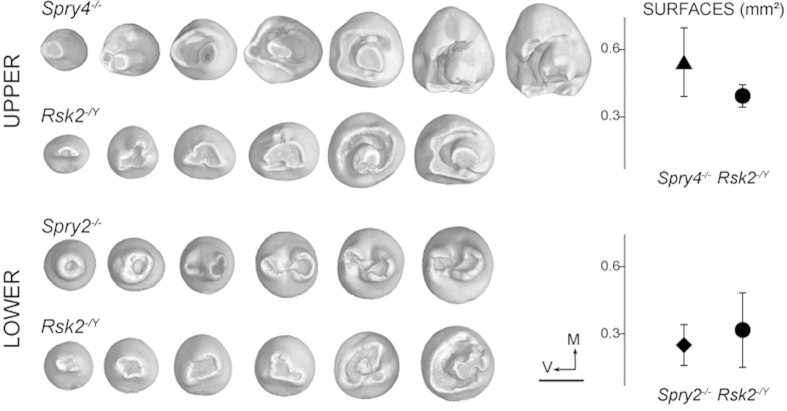
ST phenotype and surface in *Spry2*^*−/−*^, *Spry4*^*−/−*^ and *Rsk2*^*−/Y*^ mutants. ST range from small rounded monocuspid teeth to large multicuspid teeth. Right column indicates the mean surface in all the ST-displaying mutant mice, error bars represent the standard deviation. V and M respectively point towards the vestibular and mesial directions. Scale bar: 0.4 mm.

The size and shape of ST range from small rounded monocuspid teeth to large complex multicuspid teeth that can comprise up to five cusps (Fig. 5). In complex upper ST, a large central cusp is always present surrounded by a variable number of cusps linked by an almost circular crest. The most complex upper ST tend to have a mesial chevron pointing mesially (especially in *Spry4*^−/−^) whereas the lower ST mainly have a bicuspid shape (especially in *Spry2*^−/−^). These features show that ST have a clear murine shape identity.

The larger the ST is, the more the mesial parts of the neighbouring M^1^ and M_1_ are impacted. The occurrence of a ST leads to compression and flattening of the mesial part of the following teeth. This is particularly visible on M^1^ of *Spry4*^*−/−*^, and on both M^1^ and M_1_ of *Rsk2*^*−/Y*^ mutants. As a consequence, the overall occlusal area of the molar row is smaller in mutants displaying a ST, with the exception of the *Spry4*^*−/−*^ upper row, in which the area of the M^3^ seems to increase in compensation for the decrease of M^1^ area (Fig. 3).

In the most severely impacted phenotypes, the mesial contour of the M^1^ becomes rounded, whereas it is triangular in WT mice (e.g. Fig. 4e without ST). In addition, the two most mesial cusps of the first chevron become extremely reduced into a simple crest (53% *Spry4*^−/−^ and in *Rsk2*^*−*/*Y*^), and the mesio-vestibular cusp may completely disappear (20% *Spry4*^−/−^ and 4% in *Rsk2*^*−*/*Y*^). Interestingly, the mesial shrinkage of the M_1_ is associated with a change in the tilt angle of both cusps and roots of the first chevron (Suppl Fig. 1b). In WT mice, the slope of the M^1^ mesial root is in continuity with the tilt of 50° that makes the central cusp of the first chevron with the dental neck. In mutants having a ST, as well as in some mutants that do not display any ST, the tilt between the root axis and the tooth neck tends to be more vertical (about 70°), as does the slope of the first chevron central cusp (about 60°). The same type of defects can be seen when M_1_ is preceded by a ST in the three mutants: the first loph of the M_1_ is shortened and cusps appear as crushed by the presence of the ST.

## Discussion

### Similar yet distinct dental phenotypes in Sprouty and *Rsk2* mutants

Despite some variations in the penetrance of the dental defects in the mutants that we studied, which are likely to be accounted for by differences in the genetic background of our cohorts, some trends stand out. Morphological comparisons show that the occurrence of a supplementary cusp at the distal extremity of the M^1^ can be considered as a phenotypic signature of the Sprouty mutant dentitions, and *Rsk2* mutants never develop this supplementary cusp. These comparisons also highlight that *Spry4*^*−/−*^ and *Rsk2*^*−/Y*^mutants share many phenotypic features, such as the occurrence of upper and lower ST as well as abnormal arrangements in the mesial parts of the first molars. Although mesial abnormalities in these teeth may result from the occurrence of ST, a question remains concerning the higher frequency of modifications of the M^1^ first chevron in *Spry4*^*−/−*^ and *Rsk2*^*−/Y*^mutants (66–71%) when compared to the frequency of occurrence of ST (14–17%). It has already been suggested in *Eda*^*Ta*^ mutant mice that a supplementary dental germ can develop mesially to the M^1^ until advanced stages of odontogenesis without necessarily giving rise to a mineralized tooth^32^. In these cases, the development of a supplementary dental germ may be sufficient to cause disorders in the development of the mesial part of the following tooth. We can thus infer that about 70% of the specimens develop an upper ST germ that will impact on the phenotype of the M^1^.

### Roles of Sprouty and *Rsk2* genes within the ERK-MAPK cascade

Sprouty genes are thought to encode proteins that inhibit signalling through the FGFR pathway^18^,^20^,^33^. Examination of the dental phenotypes in mutant mice led to the discovery of shared features. All three Sprouty mutants share the presence of a supernumerary distal cusp, arguing in favour of shared functions. But distinct characters were also observed in each mutant, pointing to some specific functions of each of these genes in tooth morphology. The phenotypic diversity in the Sprouty mutants may be ascribed to differences in spatial and temporal expression patterns of these genes. Our data complement previous findings concerning the generation of similar phenotypes with the loss of function of *Spry2* and *Spry4* genes. *Spry4*^*−/−*^ phenotype penetrance was previously reported as lower than the one in *Spry2* null mutants^7^. Our results show that the differences between the two proportions are even larger than previously reported. This trend is reversed for the upper rows, for which we did not observe ST in *Spry2*^*−/−*^ mice, whereas these were found in 17% in *Spry4*^*−/−*^ mice. The proportion of ST in mutant dental rows was comparable to what has been recently reported^22^, but lower than the first publication on the topic^7^. Such a change in penetrance could be explained by a shift in the genetic background over generations. Finally, neither of the previous reports^7^,^22^ studied the effect of loss of function of *Spry1*. Our work shows that *Spry1*^*−/−*^ mice also display modifications in the shape of the M^1^ and M^2^, as well as in overall size of the dental row.

RSK2 is an effector kinase that acts downstream of the MAPK cascade, whereas Sprouty genes modulate the cascade through their inhibitory effect on the pathway. The possible negative feedback regulation from *Rsk2* on the adaptor protein SOS, which recruits Ras^27^, may lead to similarities in the phenotypes of Sprouty and *Rsk2* mutants. Both *Spry4* and *Rsk2* are expressed during odontogenesis in the dental papilla^8^,^21^,^34^,^35^, which may explain the high degree of similarity in the phenotypes associated with their loss of function. Our study thus provides an example of molecular and phenotypic convergence of various steps in the same signalling pathway.

Mutations in Sprouty genes and *Rsk2* lead to more teeth and more cusps. This is the opposite trend of mutations in the Fgf genes (e.g. mutations in *Fgf3*^36^,^37^ lead to fewer cusps). This contrast in phenotypic impacts of the mutations between *Rsk2* and *Fgf3* is concordant with the current knowledge of regulatory feedbacks acting on the whole pathway. Overall, in terms of phenotype penetration, one must note that the frequencies depend on the defect considered, and that the penetrance is never complete. Variations in the initial genetic background of each line might be responsible for some of the differences observed. Structural conservation and functional redundancy of the actors of the pathway^38^ can however account for the shared dental defects.

### Molecular basis of ST development

In addition to Sprouty and *Rsk2* null mice, other mutants have been described with ST mesial to the first molars. Since some of these mutations are in genes encoding proteins that interact with the ERK-MAPK pathway, this pathway appears to be a central actor. The balance between SHH and Wnt signals has been demonstrated to be crucial for the development of the diastemal rudimentary tooth germ. The link between the two pathways involves regulation of expression of Fgf pathway members by the Wnt signalling pathway. Mice mutant for *Gas1*, *Shh*, *Wnt1*^23^, *Wise*^39^, *Lrp4*^40^, *Apc*, *Ctnnb1*^41^, *Eda* or *Edar*^42^ genes are characterized by a disruption of this important molecular equilibrium and display ST. Apart from the *Apc* and *Ctnnb1* null mutants, the ST occurs very similarly to what is observed in Sprouty and *Rsk2* mutants. Thus, modulation of Wnt signalling upstream of the ERK-MAPK pathway could mimic the effect of loss of Sprouty gene or *Rsk2* function, which might explain phenotypic similarities in the occurrence of ST in both pathways.

The *Apc* and *Ctnnb1* null mutants display many more ST located in the vicinity of the incisor region^41^. These authors hypothesized that incisor stem cells might be migrating, thus generating odontogenesis-competent *foci*. The difference between one ST being displayed in a mesial position (compared to the molar row), and multiple ST being displayed in a disordered manner is likely influenced by the direct interaction with Ras and ERK. Indeed, such interactions have been demonstrated in the context of multiple cancer models in the mouse^43^,^44^.

### Potential roles of Sprouty and *Rsk2* genes in the evolution of rodent dentition

The most striking phenotypic trait of *Spry2*^−/−^, *Spry4*^−/−^ and *Rsk2*^*−*/*Y*^ mutants is the occurrence of ST located in front of the first molars at both the lower and the upper jaws. It has long been known that the rodent dentition has evolved towards a reduction in the number of teeth over the course of evolution in parallel with a specialization of the molar teeth^45^. Basal rodents had lower and upper premolars, and it is only since the rise of muroid rodents that premolars have been completely lost in the lineage leading up to the murine rodents. It has however been demonstrated that rudimentary dental germs are maintained in both the upper and the lower diastema during mouse early dental development^24^,^25^. Because these rudimentary germs are located in the vicinity of the first molar germs, they have been considered to be rudiments of premolar germs lost over evolution^26^. These rudimentary germs rapidly abort by apoptosis and do not become autonomous mineralized teeth. It has been shown that they can contribute to the development of the M_1_ by merging with the M_1_ germ^26^. Although it has not yet been formally proved, it is likely that the same mechanism occurs with the upper rudimentary germ immediately adjacent to the M^1^^46^ for participation in the development of the first chevron.

**Figure 6:**
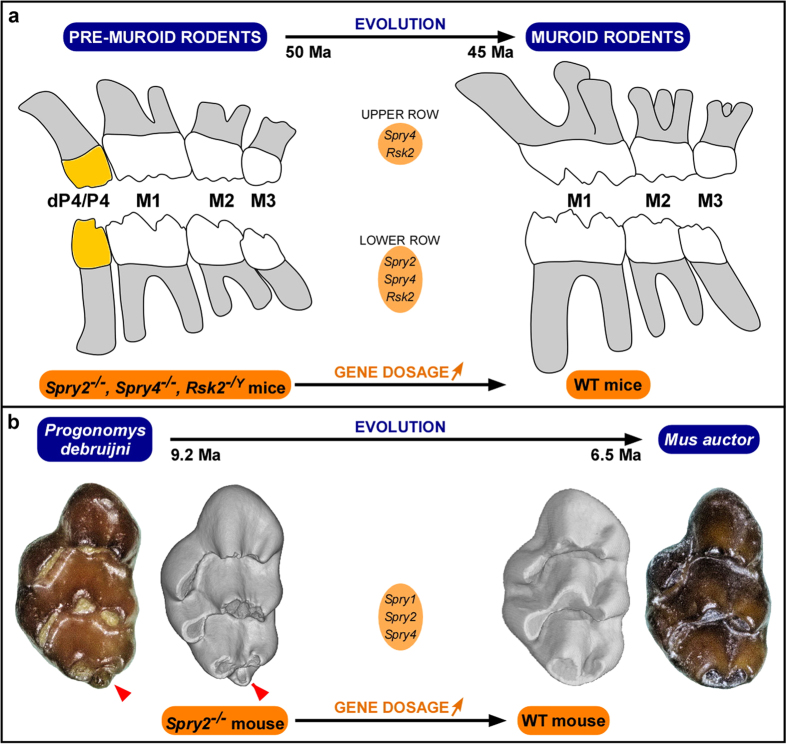
Mutant dentition recapitulates the evolution of murine tooth characters. (**a**) The dental phenotype arising from the loss of function of *Spry2*, *Spry4* and *Rsk2* genes mimics the reduction of tooth number in muroid rodent evolution. (**b**) Sprouty M^1^ mimic *Progonomys debruijni* M^1^, as depicted with the example of a *Spry2*^*−/−*^ M^1^. *M. auctor* and *P. debruijni* molar pictures are courtesy of Y. Kimura.

Here we deliver new insights into the role of the MAPK signalling pathway members beyond what has been previously reported^7^,^8^,^22^. Interesting, this signalling pathway is conserved in mammalian species, and its signal transduction properties are even shared more broadly^38^,^47^, adding its plausible role in the evolution of mammalian dentition. Here, the loss of function of *Spry2*, *Spry4* or *Rsk2* allows the continuation of the development of some rudimentary premolar germs until the formation of autonomous mineralized teeth. The segmentation of the postcanine dental row is thus impacted, because it comprises four teeth instead of three and the mesial parts of the first molars are accordingly reduced (Fig. 4). We can thus assign the ST located immediately in front of the first molars to deciduous fourth premolar teeth (dP4 and dP_4_) that will not be replaced. Teeth located at the fourth premolar position are known in the earliest muroid rodents^48^ as well as in some extant dipodoids such as *Sicista* and *Zapus*^49^,^50^, the sister group of muroids. Fourth premolars are present in the squirrel- and in the guinea pig-related clades, which arose earlier in the history of rodents than the mouse-related clade^51^. The loss of function of *Spry2*, *Spry4* or *Rsk2* involves developmental mechanisms that revitalize autonomous premolar teeth and that simplify accordingly the mesial parts of the first molars. The dental phenotype in *Spry2*^−/−^, *Spry4*^−/−^, and *Rsk2*^*−*/*Y*^ mutants could thus be considered as a partial reversal in the evolutionary trends of the dental phenotype through the transition from pre-muroid to muroid rodents (Fig. 6a), which is documented within the fossil record to have probably occurred between 50 and 45 million years ago (Ma)^48^,^52^.

As discussed above, many other mouse mutants display ST mesially to first molars, but both the phenotypic and the developmental aspects of the postcanine row segmentation have solely been scrutinized in mutants of the EDA pathway^32^. From these studies, it is clear that dental phenotypes in *Eda*^*Ta*^ (Tabby) and *Edar*^*dl-J*^ (Downless) mice radically differ from those of *Spry2*^−/−^, *Spry4*^−/−^, and *Rsk2*^*−*/*Y*^ mice. *Eda*^*Ta*^ and *Edar*^*dl-J*^ mice have postcanine teeth with simplified occlusal patterns characterized by the absence of many cusps, as well as by abnormal arrangements of the lophs and the chevrons respectively in the lower and upper molars^32^. In contrast, *Spry2*, *Spry4*, or *Rsk2* losses of function provoke rearrangements of the M^1^ and M_1_ mesial parts, but minimally impact the rest of the dental rows. From these arguments, we propose that *Spry2*, *Spry4* or *Rsk2* are candidate genes for a major role in the evolutionary trend towards reduction of the dental formula in muroid rodents 45 Ma.

Interestingly, a character that stands out from the mutant mice is the occurrence of a supplementary distal cusp, named the posterocone, on the M^1^ of the three Sprouty mutants (arrowhead in Fig. 6b). The posterocone is rarely present in extant species of murine rodents, whereas this cusp or the equivalent crest located at the same location, the posteroloph, were almost always present in the basal fossil murine genera such as *Potwarmus*, *Antemus*, and *Progonomys*. However, neither the modern mouse (*Mus musculus*) nor its ancestor (*Mus auctor*) displays an individualized posterocone (Fig. 6b). In the lineage leading to the modern mouse, the posterocone has been lost during the transition from *Progonomys debruijni* to *Mus auctor*, and this transition is documented to have occurred between 9.2 and 6.5 Ma by the fossil record in the Siwaliks of Pakistan^53^. Apart from the presence of a posterocone, *Spry1*^*−/−*^*, Spry2*^*−/−*^, and *Spry4*^−/−^ mice share other dental features with these basal murine genera such as the trend of the lingual cusps of the M^1^ to be less connected to the central cusps. All of these similarities indicate that *Spry1, Spry2,* or *Spry4* could have played a major role during the transition from *Progonomys* to *Mus*, which constitutes a major transition in the history of murine rodents.

## Concluding Remarks

*Spry1, Spry2, Spry4,* and *Rsk2*, which encode components of the ERK-MAPK signalling pathway are identified here as potentially major actors in the evolution of the dentition in rodents. These genes may have played a pivotal role in the reduction of the dental formula and in the rearrangement of the mesial parts of the first molars during the transition from pre-muroid to muroid rodents prior to 45 Ma. They also may have acted in the disappearance of the posterocone and in the connections of the lingual cusps to the chevrons during the transition from basal murines to *Mus auctor* between 9.2 and 6.5 Ma. Both morphological transformations happened independently, separated by about 35 Ma, suggesting that the premolar loss might have been triggered by *Rsk2* gene dosage augmentation. The posterocone would then have been lost because of an increase in Sprouty gene expression. An interesting issue remains open. Indeed the potential role of Sprouty or Fgf genes in the morphogenesis of the connections between lingual cusps and central cusps of the M^1^ chevrons needs further investigations. Here we report that Sprouty mice, especially *Spry2*^*−/−*^ and *Spry4*^*−/−*^ mice, often have M^1^ with disconnected lingual cusps. As Sprouty and Fgf genes are known to play antagonist roles during tooth morphogenesis, it is interesting to note that the loss of function of the two antagonist results in an equivalent impact on the lingual cusps of the M^1^. The role of this central pathway could be further studied using other genetically engineered mice, notably over-expressing the same molecules to understand the plausible evolutionary implications of dosage modulations. Focusing on the molecular redundancy allowing vestigial tooth buds to develop into fully erupted teeth is likely to bring new insights into the molecular networks involved in both tooth development and evolution.

## Material And Methods

### Sprouty mutant mice

All the Sprouty mutant mice were generated in backgrounds resulting from crossing between several lineages. The three mutants and the wild type mice were generated by breeding at UCSF. The sample set was composed of homozygous mice as indicated: 25 *Spry1*^*−/−*^, 50 *Spry2*^*−/−*^, 50 *Spry4*^*−/−*^, and 60 WT individuals (littermates of the various mutants). For each specimen, left and right, upper and lower tooth rows were studied independently. The age of the specimens ranged from 1 month to 2.5 months. Animal experimentation was carried out in compliance with the policies and procedures established by the UCSF Institutional Animal Care and Use Committee.

### *Rsk2* ^*−/Y*^ mice

The *Rsk2* mutant mouse line was generated as previously described^54^. Since *Rsk2* is located on the X chromosome, analyses were performed on *Rsk2*^*−/Y*^ males, on a C57BL/6J background. The sample set was composed of 45 *Rsk2*^*−/Y*^ and 45 WT littermates (*Rsk2*^*+/Y*^). Mouse protocols were complied with the 2010/63/UE directive and the 2013/02/01 French decree, and were thus approved by the CERBM-GIE: ICS/IGBMC Ethical Research Board.

### Observation and imaging of dental rows

All the heads were prepared in order to remove all non-mineralized tissues to allow good observation and measurement of the dental rows. They were examined and photographed using a Leica stereomicroscope. The measurements were obtained by following the outline of each tooth from the photos of occlusal view of the row. Thus, the length, width, and area of the tooth were produced by Leica software. Some Sprouty mutant dental rows were imaged using X-ray-synchrotron Radiation Facility (ESRF, Grenoble, France), beamline ID 19 and BM5, with a monochromatical beam at energy of 25 keV. X-ray synchrotron microtomography has been demonstrated to bring high-quality results for accurate imaging of small teeth^55^. A cubic voxel of 7.46 μm was used. Some *Rsk2*^*−/Y*^ mutant dental rows were imaged using the X-ray cone-beam computed microtomography with a Nanotom machine (GE) with a source tension of 100 kV with a cubic voxel of 3 μm. All 3D renderings were performed using VGStudiomax software.

### Statistics

Student’s t-tests were used to verify the significance of differences in tooth size between the mutant mice but also between the mutant and the WT mice. A threshold value of 0.05 (*p-value*) was used to assess the significance of the observed differences.

## Additional Information

**How to cite this article**: Marangoni, P. *et al.* Phenotypic and evolutionary implications of modulating the ERK-MAPK cascade using the dentition as a model. *Sci. Rep.* **5**, 11658; doi: 10.1038/srep11658 (2015).

## Supplementary Material

Supplementary Information

## Figures and Tables

**Figure 1:**
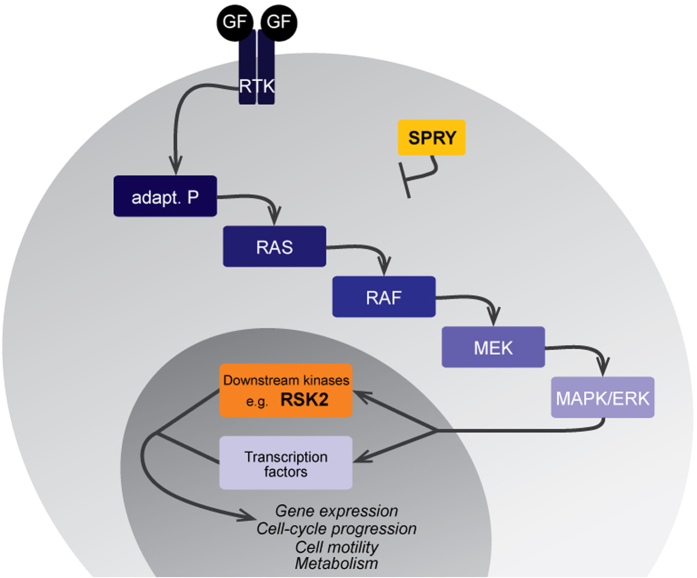
The FGF-activated ERK-MAPK cascade. The phosphorylation cascade is activated by the fixation of a growth factor to its receptor tyrosine kinase, and results in the activation of effector kinases and in the transcriptional activation of target genes. Molecular actors of this pathway that are focused on here are depicted in yellow (SPRY) and orange (RSK2).
