# Supplementary material for: Phenotypic and evolutionary implications of modulating the ERK-MAPK cascade using the dentition as a model
Source: Sci Rep. 2015 Jun 30;5:11658. doi: 10.1038/srep11658 (PMC4485067; doi:10.1038/srep11658)
Supplement: Supplementary Information [file srep11658-s1.pdf]

Phenotypic and evolutionary implications of modulating the ERK-MAPK cascade using the dentition as a model

Pauline Marangoni[1§], Cyril Charles[1§], Paul Tafforeau[2], Virginie Laugel-Haushalter[3], Adriane Joo[4], Agnès Bloch-Zupan[3,5,6], Ophir D. Klein[4,7], and Laurent Viriot[1*]

Suppl. Fig. 1: Details of some shared mutant features depicted in the *Rsk2*$^{-/Y}$ mice.

(a) The disconnection of the lingual-most cusp of the M1 1st chevron. (b) Variation of the M1 mesial root inclination in mutant mice. Note the straightening of the slope in *Rsk2*$^{-/-}$ mutants. Yellow angle is 50° in WT mice, and 60° in mutant mice. The root orientation itself is also modified, as depicted in orange.

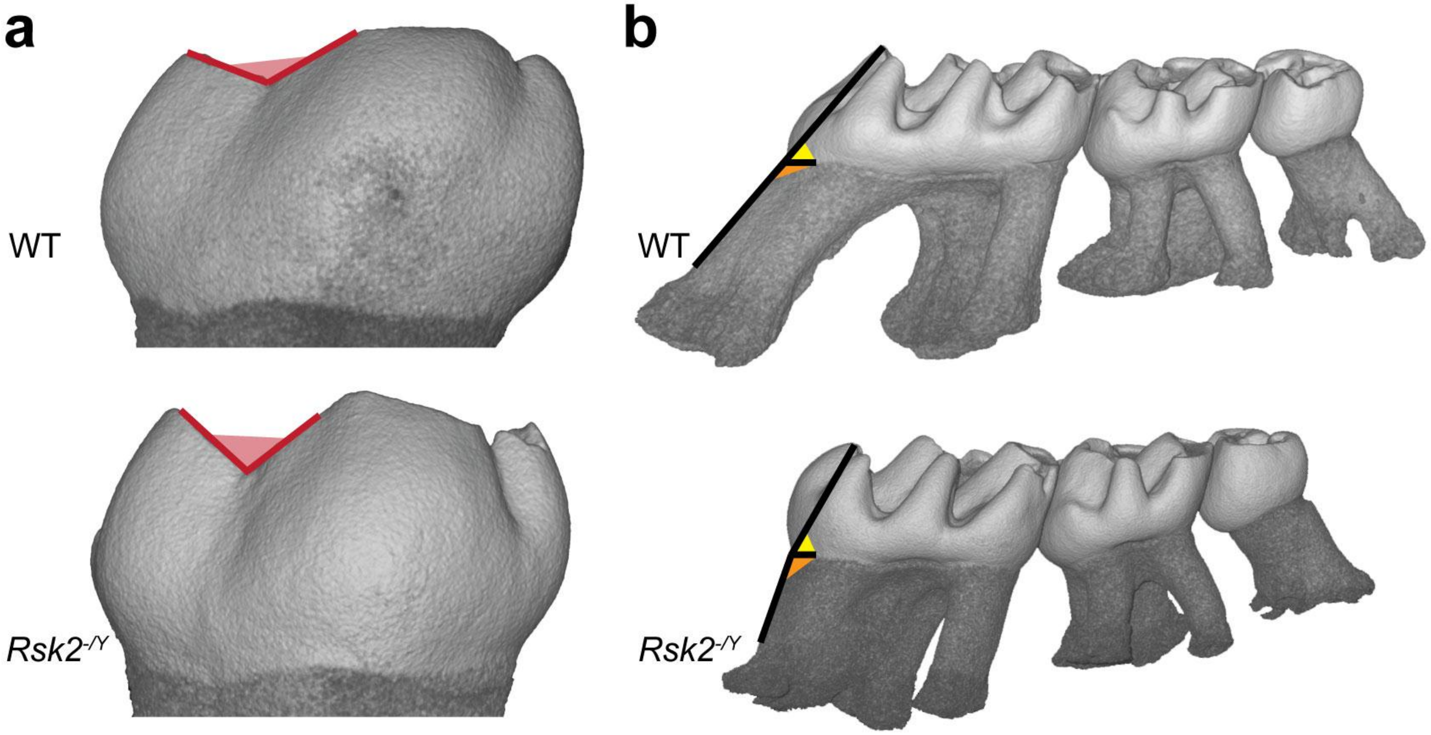
a
b
WT
WT
Rsk2-/Y
Rsk2-/Y
